# Memory of social experience affects female fecundity via perception of fly deposits

**DOI:** 10.1186/s12915-022-01438-5

**Published:** 2022-10-31

**Authors:** E. K. Fowler, S. Leigh, W. G. Rostant, A. Thomas, A. Bretman, T. Chapman

**Affiliations:** 1 School of Biological Sciences, University of East Anglia, Norwich Research Park, Norwich, NR4 7TJ UK; 2 School of Biology, Faculty of Biological Sciences, University of Leeds, Leeds, LS2 9JT UK

**Keywords:** Phenotypic plasticity, Conspecifics, Heterospecifics, *Drosophila melanogaster*, Fecundity, Cues

## Abstract

**Background:**

Animals can exhibit remarkable reproductive plasticity in response  to their social surroundings, with profound fitness consequences. The presence of same-sex conspecifics can signal current or future expected competition for resources or mates. Plastic responses to elevated sexual competition caused by exposure to same-sex individuals have been well-studied in males. However, much less is known about such plastic responses in females, whether this represents sexual or resource competition, or if it leads to changes in investment in mating behaviour and/or reproduction. Here, we used *Drosophila melanogaster* to measure the impact of experimentally varying female exposure to other females prior to mating on fecundity before and after mating. We then deployed physical and genetic methods to manipulate the perception of different social cues and sensory pathways and reveal the potential mechanisms involved.

**Results:**

The results showed that females maintained in social isolation prior to mating were significantly more likely to retain unfertilised eggs before mating, but to show the opposite and lay significantly more fertilised eggs in the 24h after mating. More than 48h of exposure to other females was necessary for this social memory response to be expressed. Neither olfactory nor visual cues were involved in mediating fecundity plasticity—instead, the relevant cues were perceived through direct contact with the non-egg deposits left behind by other females.

**Conclusions:**

The results demonstrate that females show reproductive plasticity in response to their social surroundings and can carry this memory of their social experience forward through mating. Comparisons of our results with previous work show that the nature of female plastic reproductive responses and the cues they use differ markedly from those of males. The results emphasise the deep divergence in how each sex realises its reproductive success.

**Supplementary Information:**

The online version contains supplementary material available at 10.1186/s12915-022-01438-5.

## Background

Phenotypic plasticity (the expression of different phenotypes from the same genotype) is a widespread and important component of fitness, allowing individuals to adaptively alter their behaviour or physiology in response to environmental variation [1]. An organism’s social surroundings (e.g. the local density and ratio of male  and female conspecifics and heterospecifics) can vary considerably [2]. Sex differences in birth and death rates or sexual maturity can cause temporal shifts in sex ratio, either on an immediate, short-term basis or over seasons or successive years. Other factors such as immigration, dispersal and the level of predation also contribute to a dynamic social environment [2]. The density and identity of individuals in the social milieu can signal resource quality or the expected likelihood of competition [3]. For example, the sex ratio of conspecifics could indicate the level of competition for mating opportunities or for sex-specific resources such as oviposition sites. Detection of information from heterospecifics may also be beneficial if habitat requirements overlap between species. If this is the case, the overall density of individuals, independent of species, could signal expected levels of nutrient availability or quality, predation risk or oviposition sites [4]. Given that variation in the social environment can have significant effects on reproductive competition and resource availability, individuals with the ability to detect cues that reliably indicate their social environment, and adjust their phenotype accordingly, will increase their fitness [5].

The effect of the social environment on phenotypic plasticity in males has been well-studied in the context of sperm competition [6–9]. *Drosophila melanogaster* fruitflies provide a tractable model in this context. Males can precisely and flexibly adjust their ejaculate composition and extend copulation duration in response to the presence of conspecific rival males [9–11]. These plastic adjustments enable males to secure a greater share of the paternity when sperm competition is perceived to be high, while conserving costly resources when sperm competition is unlikely [5].

There are extensive studies into male social plasticity; however, we know much less about the corresponding context in females—i.e. whether sex exposure represents a potential for increased sexual or resource competition, whether and how females respond and what cues they might use. There is a particular gap in terms of understanding the effect of prior social exposure on subsequent mating behaviour and reproductive investment—i.e. whether females have a social memory, as is found in males [5]. Findings from other insects show examples of social memory being retained, which suggest that this phenomenon could be important. For example, female cowpea weevils (*Callosobruchus maculatus*) respond to high adult densities by subsequently laying larger eggs. The larvae that emerge from these eggs make wider tunnels through the food substrate, which could give them a competitive advantage over smaller conspecifics [12]. In addition, egg and clutch size is altered if individuals of several *Daphnia* species are exposed to chemical cues of con- or heterospecifics during development [13]. In *Drosophila*, naïve females can exhibit social learning and adjust their oviposition site preferences to match those of experienced mated females [14]. Oviposition preferences can be influenced both by pheromonal cues from conspecifics [15–17] and the presence of predators [18]. Female social plasticity has also been considered in the context of mate choice and differential responses to male characteristics [19–22]. Interestingly, a recent study showed that the distribution of oviposition resources and social environment can interact to affect oviposition decisions in *D. melanogaster* females [23], which supports the idea that the responses to the social environment represent a key determinant of a female’s reproductive success.

For fitness benefits of phenotypic plasticity to be accrued by either sex, and plasticity itself to evolve, mechanisms for the accurate perception of cues that reliably indicate the social or sexual environment are required. In male *D. melanogaster*, cues of competition are detected via multiple, interchangeable olfactory, auditory and tactile sensory pathways [24]. This multimodal strategy is predicted to decrease the risk of costly mismatches between environment and phenotype in highly variable environments [6] enabling males to accurately perceive information on the species, sex and prevalence of other individuals, and respond appropriately to the level of sperm competition [25]. Whether females deploy any such multimodality via complex cues is also not yet known.

Here, we address these omissions by testing the hypothesis that *D. melanogaster* females plastically adjust their reproductive investment according to the intrasexual social environment they experience prior to mating. Focal females were either housed in isolation or with three other females before being given the opportunity to mate with a single male. We recorded mating times and the number of eggs (fecundity) laid in the 3 days before and in the 24h after mating. During the social exposure phase, all females were virgins. This allowed us to test the response of females to the same-sex environment without the confounding effects of previous mates or male pheromones. We thus investigated the effect of the social environment on current reproductive investment (virgin egg laying) and whether this social memory was carried forward into post-mating fecundity responses. We probed the underpinning mechanisms involved by varying social exposure time and by restricting the perception of social cues by using genetic and physical manipulations.

## Results

### Female fecundity responses to variation in the pre-mating social environment and effect of exposure to con- vs heterospecific females

**Fig. 1:**
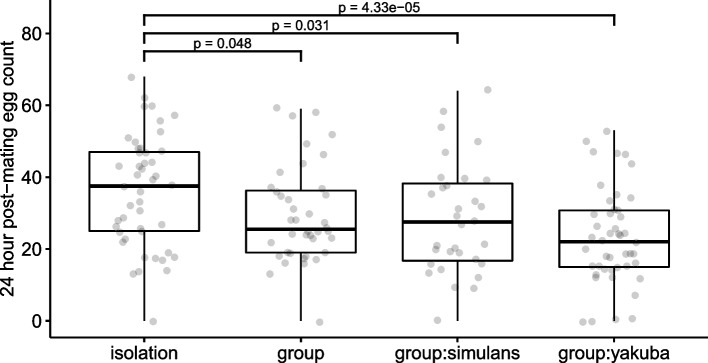
*D. melanogaster* females exposed to con- or heterospecific females prior to mating show significantly decreased post-mating fecundity. Females were kept socially isolated (‘isolation’) or exposed to con- (‘group’) or heterospecific females (‘group:simulans’ or ‘group:yakuba’) for 72h prior to mating. Fecundity was measured as the number of eggs laid by each female in the 24-h period following mating. Boxplots show interquartile range (IQR) and median in the box, and whiskers represent the largest and smallest values within 1.5 times the IQR above and below the 75th and 25th percentiles, respectively. Raw data points are plotted with a jitter. *p*-values between treatments were derived from the model summary

We measured the impact of pre-mating social isolation versus exposure to other females on the reproductive output of focal *D. melanogaster* females after a single mating. Virgin focal females were exposed to different social environments for 72h prior to mating, and fecundity was measured as the number of eggs laid in the 24-h period following mating. Overall, the social treatment had a significant effect on the number of eggs laid (Fig. 1; *F*_3,160_ = 6.10, *p* < 0.001). During the post-mating period, focal females held alone before mating laid 18% more eggs than those grouped with conspecific females, 21% more than those grouped with *D*. *simulans* females and 36% more than focals grouped with *D*. *yakuba* females (Fig. 1; *p* < 0.05; Additional file 1: Table S1).

### Effect of length of social exposure period on post-mating fecundity

**Fig. 2:**
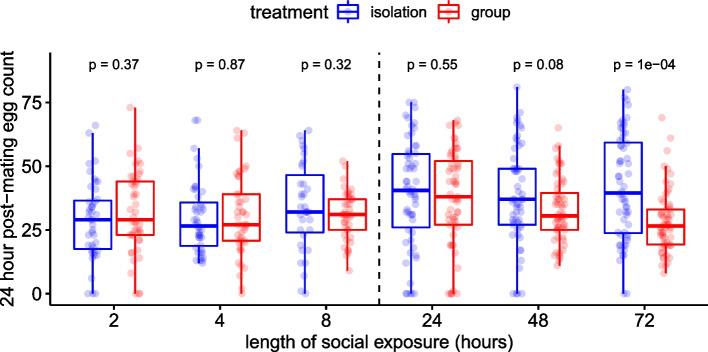
*D. melanogaster* females require 72h of pre-mating exposure to conspecifics to express fecundity plasticity. Females were housed in ‘isolation’ (blue) or in ‘group’ (red boxes) treatments, for between 2 and 72h prior to mating. The vertical dotted line indicates the data are from two separate experiments—the first ‘short-term’ experiment used 2-, 4- and 8-h exposure times, and a second ‘long-term’ experiment used 24-, 48- and 72-h timepoints. Fecundity was measured as the number of eggs laid in the 24-h period following mating. Statistical significance between social treatments for each timepoint was derived from post hoc tests of the model and is indicated above box pairs. Boxplots as in Fig. 1

The effect of the length of the social exposure period on female social responses was measured in two experiments. In the first, we measured the effect of short-term exposure (2, 4 or 8h) to other conspecific females and found no significant effect of social treatment, exposure length or their interaction on egg laying (Fig. 2; Additional file 1: Table S2). The second experiment measured the effect of longer-term exposure (24, 48 or 72h). In this case, there was a significant interaction between the social environment and the length of social exposure on a female’s fecundity (*F*_(2,350)_ = 3.81, *p* = 0.023). Specifically, females exposed before mating to conspecifics for 72h showed a significant reduction in post-mating fecundity, in comparison to previously isolated females (Fig. 2; *p* < 0.0001; Additional file 1: Table S2). There was a non-significant tendency for females previously exposed to other females for 48h prior to mating to subsequently lay fewer eggs than isolated females (Fig. 2; *p* = 0.08; Additional file 1: Table S2). Twenty-four hours of exposure produced no detectable effect on post-mating fecundity (Fig. 2; *p* = 0.55; Additional file 1: Table S2).

### Investigation of whether exposure to eggs or to non-egg deposits is required for socially induced fecundity plasticity

**Fig. 3:**
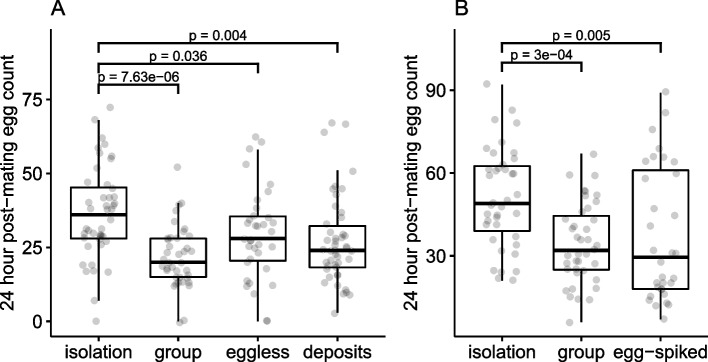
*D. melanogaster* females respond to their social environment by detecting the deposits left by other females, even in the absence of eggs. Two experiments were conducted: **A** Wildtype focal females were either isolated (‘isolation’), housed in groups of four (‘group’), housed with three *OvoD1* females (‘eggless’) or housed in vials previously occupied by three *OvoD1* females (‘deposits’); **B** wildtype focal females housed in isolation, in groups of four or in vials containing eggs laid by previous wildtype occupants (‘egg-spiked’). Fecundity was measured as the number of eggs laid by the focal female in the 24-h period following a single mating. Boxplots as in Fig. 1. Statistical significance values between isolation and other treatments were derived from model summaries

To identify the cues used by females to respond plastically to their social environment, we analysed whether a female’s post-mating fecundity responded to the presence of other females, to their eggs or to non-egg deposits following 72h of exposure. We conducted two experiments. In the first, focal females were either isolated prior to mating or exposed to 3 conspecific females, 3 *OvoD1* ‘eggless’ females, or to a food vial in which *OvoD1* ‘eggless’ females had previously been housed (i.e. contained female deposits but no eggs). Overall, there was a significant effect of the pre-mating treatment on subsequent fecundity after mating (*F*_(3,160)_ = 7.73, *p* < 0.0001; Additional file 1: Table S3). Consistent with above, females exposed before mating to conspecifics laid significantly fewer eggs after mating than did socially isolated females (Fig. 3A; *p* < 0.0001; Additional file 1: Table S3)). Furthermore, females exposed to eggless conspecifics and the deposits of eggless conspecifics prior to mating also laid significantly fewer eggs after mating in comparison to females from the ‘isolation’ treatment (Fig. 3A; Additional file 1: Table S3). In a second experiment, we tested the responses of females to the presence of conspecific eggs alone. Overall, this showed a significant effect of pre-mating environment treatment on post-mating fecundity (*F*_(2,107)_ = 8.00, *p* < 0.001), with focal females exposed to conspecific females (mean 34.5 eggs) or to conspecific eggs alone (mean 37.4 eggs) both laying significantly fewer eggs than previously socially isolated females (mean 51.2 eggs) (Fig. 3B; Additional file 1: Table S3).

### Investigation of the sensory pathways required to detect cues of socially induced fecundity plasticity

**Fig. 4:**
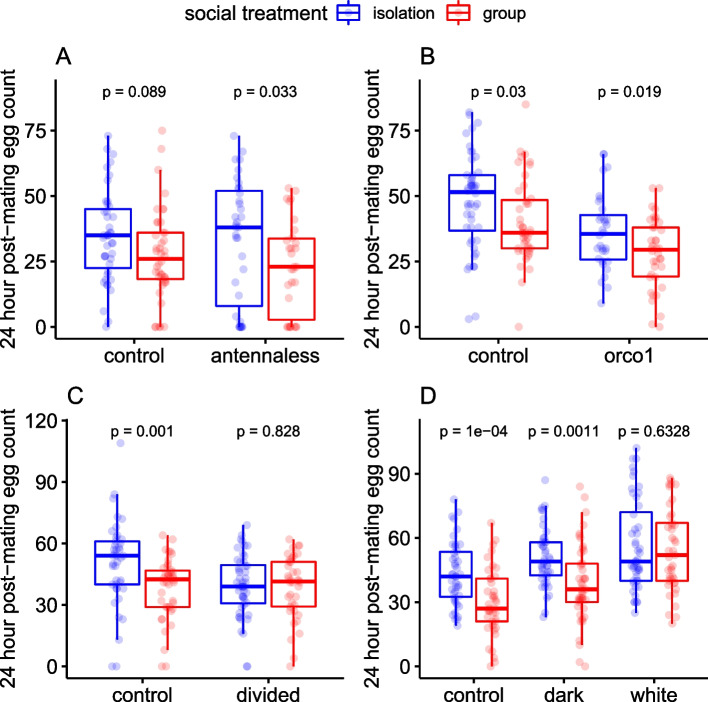
*D. melanogaster* females respond to their social environment by using tactile/gustatory sensory pathways. **A** Olfactory restriction through antennal removal. Intact focal females (‘control’) and olfactory-manipulated focal females with no third antennal segment (‘antennaless’) were kept in isolation or in a group with three intact non-focal females. **B** Olfactory restriction through *Orco* knockout. Wildtype Dahomey females (‘control’) or females lacking the general olfactory receptor Orco (‘*orco*^*1*^’) were kept in isolation or in a group with three Dahomey non-focal females. **C** Tactile/gustatory restriction. Focal females were housed in a standard vial (‘control’) or in a vial with a transparent, perforated divide (‘divided’). For the divided group treatment, focal females were physically separated from the three non-focals by the divide. **D** Visual restriction. Wildtype females held under standard light conditions (‘control’), wildtype females held in darkness (‘dark’) and *white* females (‘white’) were kept in isolation or exposed to three wildtype non-focal females. Fecundity was measured as the number of eggs laid in the 24-h period following mating. Boxplots as in Fig. 1. Statistical significance values between box pairs were derived from post hoc testing of models

To identify the sensory pathways used by focal females to detect the cues contained within non-egg female deposits, we restricted olfactory, tactile/gustatory and visual inputs in turn in four separate experiments. Each experiment included unmanipulated wildtype social isolation and group controls for comparison. To identify which sensory input was responsible, we tested in each experiment for a statistical interaction between focal female type (sensory restricted or control) and social treatment (isolation or group), with post-mating fecundity as the response variable. In the first test, we manipulated the ability of focal females to receive olfactory cues by surgically removing the third antennal segment prior to applying the social exposure treatments. We found no significant interaction between focal female type (intact/antennaless) and pre-mating social environment on post-mating fecundity (Fig. 4a; *F*_(1, 146)_ = 0.34, *p* = 0.562; Additional file 1: Table S4). Antennal removal only partially restricts olfactory sensory pathways, since a secondary olfactory system is located in the maxillary palps [26]. Therefore, to complement the antennal removal experiment, we performed a second test using focal females carrying a knockout mutation in the broadly expressed olfactory receptor, Orco, which is associated with volatile pheromone sensing [27]. As with the antennaless experiment, there was no significant interaction between focal female type (*Orco*^−^ or wildtype) and pre-mating social environment on post-mating fecundity (Fig. 4b; *F*_(1, 157)_ = 0.33, *p* = 0.564; Additional file 1: Table S4).

We next tested for the influence of tactile/gustatory cues. For this, focal females were separated from non-focals in the same vial using a perforated acetate divide. In this experiment, we found a significant interaction between focal sensory input and pre-mating social environment on post-mating fecundity (*F*_(1,165)_ = 4.21, *p* = 0.042). Post hoc tests revealed that focal females that were physically separated from conspecifics did not significantly differ in post-mating fecundity from isolated females in equivalent housing (i.e. focal females in an acetate-divided vial with *no* conspecifics in the opposite chamber), implying physical contact with social cues is required for females to express plasticity (Fig. 4c; Additional file 1: Table S4).

In the fourth experiment, we tested the importance of visual input cues, using either wildtype focal females held in darkness throughout the pre-mating social exposure period or vision-defective *white* focal females held under normal light conditions [28]. The results of manipulating visual cues in these two different ways produced inconsistent effects evident as a significant interaction between sensory input and pre-mating social environment on post-mating fecundity (Fig. 4d; *F*_(2,255)_ = 4.46, *p* = 0.012; Additional file 1: Table S4). Post hoc tests revealed that this occurred because females held in darkness retained significant post-mating fecundity responses to their pre-mating social environment whereas *white* focal females did not (Additional file 1: Table S4). We suggest that the retention of fecundity plasticity in females held in the dark suggests that vision is not the primary cue used by females. We interpret the loss of plasticity in *white* females as a potential pleiotropic effect of the *white* mutation separate from vision itself (see the ‘Discussion’ section).

### Effect of pre-mating social environment on immediate virgin egg retention

**Fig. 5:**
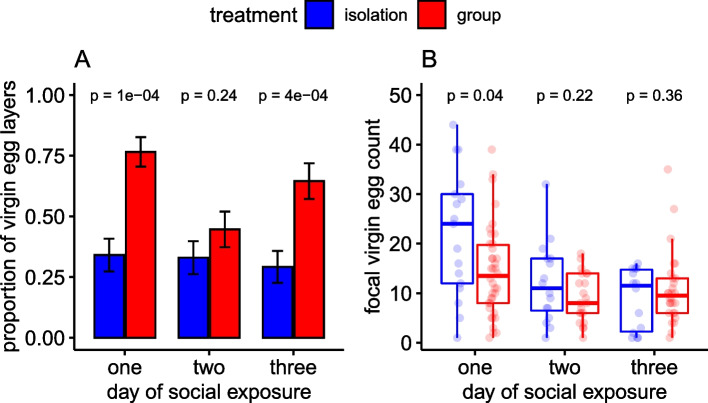
*D. melanogaster* females housed in groups are less likely to retain virgin eggs. Focal females were kept in ‘isolation’ (blue bars/boxes) or ‘group’ (housed with three Sudan Red-dyed non-focal females, red bars/boxes) treatments, for 3 days. **A** The proportion of virgin females laying ≥1 egg on days 1, 2 or 3 of social exposure. **B** Virgin egg counts of laying females (laying ≥ 1 egg on any given day) over 3 days of social exposure. Boxplots as in Fig. 1. Statistical significance values and standard error bars were derived from post hoc testing of models

**Fig. 6:**
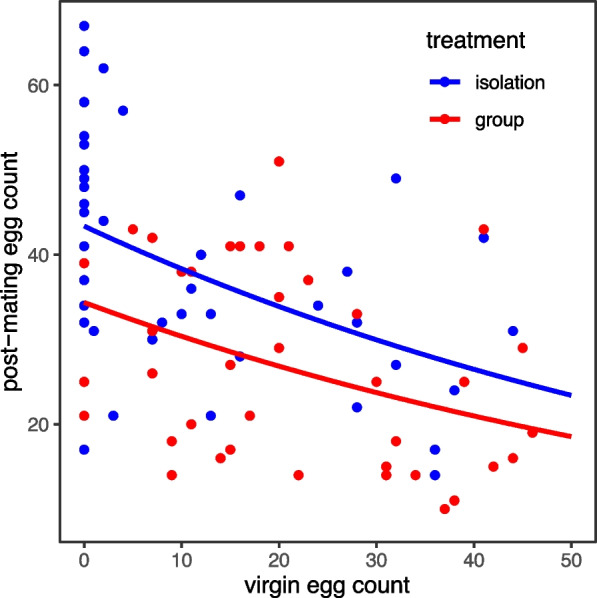
Negative relationship between pre- and post-mating fecundity in socially isolated and grouped females. Shown is the relationship between the total number of virgin eggs laid by a focal female in the 3 days prior to mating and the number of post-mating eggs laid for 24h after mating. Focal females were held in either ‘isolation’ (blue) or in ‘group’ (with three Sudan Red-dyed non-focal females prior to mating, shown in red) treatments

To test for any potential associations of pre- and post-mating fecundity plasticity, we also examined the number of virgin (unfertilised) eggs laid by isolated and grouped females prior to mating. Eggs laid by the focal female in the group treatment were distinguished from those of the non-focal by the use of an oil-based dye which was fed to non-focal females only. Non-focal eggs therefore appeared pink in colour. Virgin egg count data were zero-inflated (the expected number of zeros under a Poisson distribution was 6, and the observed was 156). Therefore, we used a two-step hurdle model to test for the effect of the pre-mating social environment on the number of virgin eggs laid by focal females over the 3 days prior to mating. Overall, there was a significant interaction between social environment and the day of social exposure on the number of virgin eggs laid by focal females (Fig. 5; *χ*^2^_(4,277)_ = 9.94, *p* = 0.04; Additional file 1: Table S5). Post hoc testing of the binomial part of the model showed that grouped females were always more likely than isolated females to lay at least one egg, and this was significant on days 1 and 3 of social exposure. Post hoc testing on the negative binomial part of the model showed that of females who laid ≥1 egg on a given day isolated females laid significantly more eggs than did grouped females on day 1 of exposure (Fig. 5; Additional file 1: Table S5). Therefore, grouped females were more likely to *start* laying eggs as virgins, but isolated females who *did* lay eggs tended to lay more than grouped females. Analysis of the fecundity of these same females after mating showed that both social environment and the total number of virgin eggs laid by focal females significantly affected post-mating fecundity. Isolated females laid significantly more post-mating eggs than previously grouped females (*F*_(1,85)_ = 7.1, *p* = 0.009), and there was a negative correlation between the total number of virgin eggs and post-mating eggs laid by a focal female (Fig. 6; *F*_(1,86)_ = 30.9, *p* < 0.0001; Additional file 1: Table S5). This was true for isolated females when egg-laying and egg-retaining females were included in the analysis or when only egg-laying females were included (Additional file 1: Fig. S1).

### Effect of pre-mating social environment on mating latency and duration

Mating latency varied significantly with the pre-mating social environment in the control groups in five of the nine experiments (Additional file 1: Fig. S2, Table S6). In those five cases, previously grouped females were always slower to mate than isolated females. Mating duration did not vary with pre-mating social treatment in eight of the nine control experiments (Additional file 1: Table S7). The exception was the 72-h timepoint from the ‘length of social exposure’ experiment in which previously grouped females had a significantly shorter mating duration than isolated females (Additional file 1: Fig. S3).

## Discussion

We found that female post-mating fecundity varied according to the pre-mating intrasexual social environment. Females exposed prior to mating to groups of con- or heterospecific females showed significantly reduced post-mating fecundity in comparison to socially isolated females. Between 48 and 72h of exposure was required for post-mating fecundity to develop this significant plasticity. Direct contact with deposits previously left by other females was sufficient to initiate fecundity plasticity, suggesting that the relevant cues are detected using tactile or gustatory pathways. Virgin egg retention was significantly higher among isolated in comparison to grouped females, and there was a negative relationship between virgin and post-mating fecundity, regardless of social treatment. The results show that females can retain and respond to the memory of their previous social environment by detecting the non-egg deposits of other females. The socially induced plasticity we have identified here contrasts markedly with that of males.

### Female fecundity varies plastically according to the pre-mating social environment

The results reveal that the imprint of the pre-mating social environment is retained, and significantly affects post-mating fecundity, consistent with findings by Churchill et al. [23]. Such plasticity is expected to have profound fitness consequences for both the female experiencing the social environment and her mate. Females responding to others in their pre-mating environment may gain benefits by optimising fecundity responses according to the expectation of oviposition sites and food availability. The presence of other adults and larvae at oviposition sites is known to have a significant impact on larval survival. Higher adult densities at oviposition sites lead to increased larval survival [29, 30]. However, very high larval densities create competition and also lead to a lower larval survival rate [30]. Therefore, a potential benefit of fecundity plasticity might also be for females to adjust their oviposition rate according to the expected larval density and therefore optimise the survival of offspring by avoiding over-crowded or under-populated developmental conditions. The pattern we observed is consistent with potential benefits for grouped females in avoiding competition at oviposition sites, by laying fewer eggs, and for isolated females to achieve density-dependent benefits by laying more. It is also possible that females could benefit from fecundity plasticity in order to benefit explicitly from the production of public goods. For example, in grouped situations, females might calibrate their fecundity to the level where they optimise benefits from the amount of tunnelling in the food medium and production of diffusible antimicrobials or anticannibalistic molecules on the surface of eggs [31, 32]. This is consistent with previous research which found that oviposition can vary as a function of adult density [33]. Another explanation for previously grouped females laying fewer eggs after mating could be that they trade off offspring quantity for quality in environments where they expect their offspring to be in competition.

Interestingly, fecundity plasticity was not restricted to the conspecific social environment, as exposure of *D. melanogaster* females to either *D. simulans* or *D. yakuba* females prior to mating also resulted in significantly reduced post-mating fecundity. *D. simulans* and *D. yakuba* are both members of the *melanogaster* species subgroup and show geographical overlap. All three species are also generalists, requiring rotting fruit for oviposition [34]. Sensory cues such as chemical or pheromonal are already known to be shared across closely related species. For example, aggregation pheromones across *D. melanogaster*, *D. yakuba* and *D. simulans* appear identical [35] and attract heterospecifics as well as conspecifics in the field [36, 37]. There could be benefits to individuals from responding to heterospecific cues if food or oviposition resources are shared, and thus heterospecific cues signal resource quality or expected levels of competition for potentially limited, shared resources. For example, larval food substrates may be exploited by different species, meaning that oviposition decisions based on the presence of heterospecifics could minimise over-exploitation and boost fitness [17, 30, 38]. There is increasing evidence that individuals can also ‘mark substrates’ as a deterrent effect [39]. We suggest that fecundity plasticity allows females to optimise their egg laying when oviposition and larval resources are likely to be utilised by closely related species in sympatry. Interestingly, male *D. melanogaster* respond plastically to the presence of con- and some heterospecific males (*D. simulans* and *D. pseudoobscura*) but not others (*D. yakuba* or *D. virilis*) by increasing mating duration. However, the heterospecific responses when present do not occur to the same extent as following conspecific exposure [25], likely because male responses to heterospecifics would carry costs but apparently little benefit (since heterospecifics pose minimal sperm competition). For females however, the benefits of basing oviposition decisions on the presence of sympatric heterospecifics vs conspecifics may be similar due to shared resource use [40].

### Females require between 48 and 72h of social exposure to express fecundity plasticity

Responses by females to their social environments were not instantaneous and appeared to take longer to develop than the 24h that is reported for behavioural plasticity in males [41]. The precise social environment adult flies experience in the wild is likely to be subject to rapid changes, as flies eclose, move between patchy food resources or die. Such rapid variation may not provide a reliable indication of resource levels for females, thus setting up the requirement for a longer threshold of exposure to cues before decisions about potentially costly reproductive investment are triggered. Therefore, it is likely that the types of social responses seen in this study only benefit females if the social environment is sustained and thus accurately signals resource levels. It is also possible that the development of social memory requires some minimum of learning time. We suggest that transient changes in the social environment are unlikely to represent accurate indicators of resource quality to an even greater extent for females than males [42].

### Non-egg deposits from previous vial occupants stimulate the fecundity response

Interestingly, non-egg-derived deposits left behind by other females were sufficient to stimulate post-mating fecundity plasticity. This is consistent with the observation that residual cues from either sex can influence egg placement decisions in *D. melanogaster* [16]. Cues could include pheromones or microbes deposited from the cuticle or in the insect excreta (frass). Reproductively mature, virgin females harbour 50 types of cuticular hydrocarbon (CHC) and fatty acid molecules [43]. Female frass also contains CHCs such as methyl laurate, methyl myristate and methyl palmitate, and responses to deposited frass are reported to lead to increased feeding and aggregation [44]. Chemical cues are likely to be sensed by olfactory or gustatory sensory pathways, and indeed, olfactory receptors were found to be partly responsible for behavioural changes in response to frass [44]. Frass deposits could provide a persistent and accurate indicator of the local population density and composition and thus a more accurate indicator of potential resource levels as opposed to the detection of the numbers of flies present at any given time, which could fluctuate rapidly.

### Direct contact with deposit cues is required, suggesting the use of gustation

Females that were physically separated from other flies and eggs did not differ in fecundity from isolated females. Combined with our finding that non-egg-derived female deposits are sufficient to stimulate plastic fecundity responses, these results suggest that gustatory (rather than tactile) pathways are used by females to respond to their pre-mating social environment. Previous studies have found that female flies use sensory receptors located in their legs, ovipositor and proboscis to sample egg-laying sites [45] and integrate olfactory and gustatory cues to make egg-laying decisions. Visual cues appeared not to be necessary, as fecundity plasticity was retained in females held in the dark. However, we observed that visually impaired *white* females did lose their fecundity plasticity. This could indicate that some aspect of visual input disrupted by *white* is important for this response, although pleiotropic effects of the *white* eye mutation, such as impaired memory [46], or compromised gravitaxis [47] are also potential explanations. That gustatory cues alone appear to be sufficient for females to assess and respond to social cues is in contrast to the multimodal strategy seen in males [24]. This may reflect the complexity of information required to make the appropriate response in each sex or the type of plastic phenotype involved.

### The social environment alters virgin egg retention

Isolated virgin females were more likely to retain unfertilised eggs than those held in a group. This may be an adaptive strategy to conserve resources during long non-reproductive periods [48] or when high-quality oviposition sites are unavailable. Our finding that female *D. melanogaster* are more likely to retain virgin eggs in social isolation is consistent with observations for the tephritid *Rhagolettis pomanella* [49] and may indicate that a social stimulus is required for females to initiate ovulation. A benefit of high virgin egg retention was increased fecundity following mating, consistent with previous findings [50].

### Mating behaviour was not consistently affected by social environment in females

The effect of pre-mating social exposure on mating latency was inconsistent, although when there was a significant effect, it was always that grouped treatment females were slower to mate. Interestingly, recent work by Churchill et al. also reported that grouped virgin females were significantly slower to mate than isolated females [23]. Similar inconsistency in the influence of the social environment upon male mating latency is also observed, but again when there is a significant effect, it is males exposed to conspecifics that are slower to mate than isolated males [5, 51–53].

In all but one experiment, mating duration was unaffected by a female’s previous social environment, and in the one case where there was an effect, it was that matings were shorter for females grouped prior to mating, consistent with a result reported by Churchill et al. [23]. In contrast, male *D. melanogaster* consistently show the opposite pattern and extend mating duration by several minutes when previously exposed to rival males [5], consistent with the idea that mating duration is largely under male control [51].

## Conclusions

Overall, these results show that the imprint of the intrasexual social environment prior to mating affects a female’s investment in reproduction. The mechanism for this effect depends upon the detection of non-egg female deposits, suggesting that gustation is important. The responses, timing and nature of cues used are markedly different in females vs males, reflecting the contrasting benefits of reproductive plastic behaviour between the sexes.

## Methods

### Fly stocks and handling

Wildtype *D. melanogaster* flies were from a large laboratory population originally collected in the 1970s in Dahomey (Benin) and maintained in stock cages with overlapping generations. Wildtype *D. simulans* and *D. yakuba* were obtained from the San Diego *Drosophila* Stock Center and KYORIN-Fly *Drosophila* species stock centre (stock #k-s03), respectively. Flies were reared on standard sugar yeast (SY) medium (100 g brewer’s yeast, 50 g sugar, 15 g agar, 30 ml Nipagin (10% w/v solution), and 3 ml propionic acid, per litre of medium) in a controlled environment (25°C, 50% humidity, 12:12-h light:dark cycle). For the Sudan Red food medium, 800 ppm Sudan Red 7B (*Sigma Aldrich*) dye was added to the SY diet before dispensing. Eggs were collected from population cages on grape juice agar plates (50 g agar, 600 ml red grape juice, 42 ml 10% w/v Nipagin solution per 1.1 l H_2_O) supplemented with fresh yeast paste, and first instar larvae were transferred to SY medium at a standard density of 100 per vial (glass, 75×25mm, each containing 7ml medium). Male and female adults were separated within 6h of eclosion under ice anaesthesia and stored in single sex groups of 10/vial. *White* females were from a stock carrying the *w*^*1118*^ allele that had been backcrossed three times into the Dahomey wildtype. *Orco* females were generated from backcrossing *Orco*^*1*^ (Bloomington Drosophila Stock Centre, stock #23129) stock for three generations into a Dahomey stock carrying the *TM3 Sb ry* balancer on chromosome 3. Eggless females were generated by crossing males from the *Ovo*^*D1*^ stock [54] with wildtype Dahomey females.

### Effect on female mating behaviour and fecundity of variation in pre-mating social environment

In all experiments, virgin focal *D. melanogaster* females were CO_2_ anaesthetised at 3–4 days old, pooled from across storage vials and then randomly assigned to isolation (1 female per vial) or group (1 focal and 3 virgin non-focal females per vial) social treatments. Females were exposed to these social environments for a period of 72h (unless stated otherwise) prior to mating. Wildtype males were aspirated individually into fresh SY vials the day prior to the mating trial. Mating trials were conducted at 25°C at 50% RH, always starting at 9am in the morning unless otherwise stated. On the day of mating, focal females were aspirated into vials containing a single male. Pairs were observed and the introduction time, start and end of mating were recorded. Any flies that did not start mating within 90 min were discarded. Males were removed immediately following the end of copulation and females left to oviposit for 24h before being discarded. Eggs laid on the surface of the SY medium in this 24-h period were counted under a Leica MZ7.5 stereomicroscope. Final sample sizes (number of biological replicates) for all experiments are shown in Additional file 1: Tables S1-S7 and range from 37 to 62 depending on the experiment.

### Female fecundity responses to variation in the pre-mating social environment and effect of exposure to con- vs heterospecific females

Following the protocol above, focal wildtype *D. melanogaster* females were kept in isolation or housed with 3 non-focal females of the same or two different *Drosophila* species prior to mating. We chose as heterospecific treatments two species of the *melanogaster* subgroup—*D. simulans* and *D. yakuba*, which shared their last common ancestor with *D. melanogaster* ~5 MYA and ~13 MYA, respectively [55]. Non-focal females were wing-clipped under CO_2_ anaesthesia prior to setting up the social exposure treatments, in order to distinguish them from the focal *D. melanogaster* individuals.

### Effect of length of pre-mating social exposure period on post-mating fecundity

The experiment was set up following the standard protocol above, with wildtype Dahomey focal and non-focal females, but with varying lengths of social exposure before mating. To test the effect on post-mating female fecundity from shorter-term exposure, all females were placed into the social environments in parallel (between 9 and 10am on the day of the mating trials), then subsets of focal females were mated after 2, 4 or 8h. Therefore, these matings were conducted at different times of the day (2h at 12pm, 4h at 2pm and 8h at 6pm). Longer-term exposure was tested in a separate experiment. Again, all social environments were set up in parallel, then mating trials on subsets of focal females were conducted after 24, 48 and 72h, all at 9am each day.

### Investigation of whether exposure to eggs or to non-egg deposits is required for socially induced fecundity plasticity

This experiment was carried out in two parts. In the first, we tested whether exposure to eggs of other females, or deposits of other females in the absence of eggs, was required for females to show plastic fecundity responses after mating. To do this, we used non-focal females from the *Ovo*^*D1*^ (eggless) genotype. Wildtype focal females were kept alone (isolation), exposed to 3 wildtype non-focal conspecifics (group), 3 eggless *Ovo*^*D1*^ non-focal females (group—eggless females) or an SY vial that had previously housed 3 eggless *Ovo*^*D1*^ females for the preceding 24h (isolation—female deposits). In the second set, wildtype focal females were again kept alone (isolation), exposed to 3 wildtype non-focal conspecifics (group) or exposed to eggs laid in the previous 24h by three wildtype non-focals (isolation—egg-spiked). In both experiment sets, all focal females were moved to ‘fresh’ (deposits, egg-spiked or clean food) vials every 24h of the exposure period to maintain the strength of the specific cues involved.

### Investigation of the sensory pathways required to detect cues of pre-mating social exposure effects on socially induced fecundity plasticity

To identify the sensory pathways used by females to detect female presence described above, we conducted three sets of experiments, each with standard isolation and group control treatments. To test the effect on post-mating fecundity of manipulating visual inputs, we used either wildtype females held in darkness or visually defective *white* focal females held under normal light conditions [28]. The *white* line was derived by repeatedly backcrossing *w*^1118^ into the Dahomey wildtype genetic background [56]. Non-focal females were all wildtype. To test the effect of manipulating olfactory cues, we used focal females with a knockout mutation in the *Orco* gene (encoding a broadly expressed odorant receptor, essential for olfaction of a wide range of stimulants [27]), or we surgically removed the third antennal segment of wildtype focal females under CO_2_ anaesthesia 1 day prior to setting up the social treatments. The antennal segment contains sensillae bearing odorant receptors, but also aristae that detect sound [57, 58]. Non-focal females for both olfactory experiments were wildtype females with intact antennae, which were wing-clipped under CO_2_ anaesthesia 1 day prior to social exposure. Finally, to test the effect of manipulating tactile cues, we physically separated wildtype focal females from non-focals using a perforated acetate divider to create two chambers within a standard vial. Perforations allowed the transmission of sound and odours, and the dividers were translucent which allowed for the perception of visual cues.

### Effect of social environment on virgin egg retention

In the final experiment, we used a novel egg marking procedure to test the effect of isolation and group treatments on pre-mating (virgin) egg production and retention. Wildtype focal females were reared according to the standard protocol. Non-focal females were reared from the 1st instar larval stage on SY food containing 800 ppm oil-based Sudan Red dye, which stains lipids, resulting in the production and laying of visibly pink eggs as adults. Dyed females were collected upon eclosion and maintained on Sudan Red food for 3–4 days prior to setting up the social treatments. Social treatments were set up according to the standard protocol, above. For the group treatment, one focal female was housed in a vial with three dyed non-focals. Females were then moved every 24h to fresh food until mating. The number of white and dyed (pink) eggs laid by the focal and non-focal females, respectively, was recorded for each 24-h period of social exposure. Mating trials and post-mating egg counts were conducted as above.

### Statistical analysis

Statistical analyses were carried out in R v 3.6.3 [59], using the ‘stats’ package for conducting generalised linear models (GLMs), ANOVAs of models and *t****-***tests, the ‘pscl’ package for hurdle models, the ‘survival’ package for cox proportional hazard models and ‘emmeans’ package for post hoc testing. Figures were made using ‘ggplot2’ and ‘ggpubr’ packages.

#### Experiment 1

The number of post-mating eggs was analysed using a GLM with social environment (four levels: isolated, *melanogaster*, *simulans* and *yakuba*) as the fixed dependent variable, a log link and quasi-Poisson errors to account for over-dispersion. Significance values were derived from an ANOVA of the model compared with a null model, using an *F*-test (Additional file 1: Table S1).

#### Experiment 2

This experiment was conducted in two separate parts (short-term: 2, 4 and 8h and long-term 24, 48 and 72h) and so two separate analyses were carried out. For both experiments, the number of post-mating eggs was analysed using a GLM with social environment (two levels: isolated, grouped), timepoint (three levels, as factors) and their interaction as dependent variables, a log link and quasi-Poisson errors. Models with and without the interaction term were compared using anova() and the interaction was dropped from the model if there was no significant difference between the full and reduced model. Pairwise post hoc tests were conducted on the final models using emmeans() (Additional file 1: Table S2).

#### Experiment 3

This experiment consisted of two parts, so two analyses were carried out. In each analysis, post-mating eggs were analysed in a GLM as for experiment 1, with social treatment as a fixed effect. In the first analysis, social treatment had four levels (isolation, group, female deposits and eggless), and in the second, social treatment had three levels (isolation, group, egg-spiked). Significance values were derived using an anova() as described for experiment 1 (Additional file 1: Table S3).

#### Experiment 4

For each sensory manipulation (four separate experiments and therefore analyses), the number of post-mating eggs was analysed using a GLM as above. In each analysis, we tested specifically for an interaction between social treatment (two levels: isolation, group) and sensory manipulation (two levels: intact, manipulated). In the vision experiment, sensory manipulation had three levels since there were two types of manipulation—dark and *white*. Models with and without the interaction term were compared using anova() as described for experiment 2. Pairwise post hoc tests were conducted on models containing the interaction term using emmeans() (Additional file 1: Table S4).

#### Experiment 5

The number of virgin eggs was analysed using a hurdle model, with social treatment (two levels: isolation and group), day (three levels: 1, 2, 3) as a factor and the interaction between them as dependent variables. Positive counts were tested using a truncated negative binomial with a log link, and zero counts with a binomial with logit link. Models with and without the interaction term were compared using waldtest() from the ‘lmtest’ package. Pairwise post hoc tests were conducted for each part of the hurdle model (binomial, or negative binomial) using emmeans() (Additional file 1: Table S5).

#### Mating latency and duration

Mating latency was analysed using Cox proportional hazards models, fitted using the coxph() function. Individuals that did not mate within 90 min were treated as censors. For mating duration, times of < 6 min and > 30 min were excluded from the analysis. These data points represent extremely short copulations, in which genitalia were unlikely to have been fully engaged or sperm transferred [60]. Very long copulations can result if genitalia become ‘stuck’ and flies fail to disengage. In total, 11 such outliers were removed from across five of the mating duration experiments (Additional file 1: Table S7). Mating duration data were normally distributed for each experiment (Shapiro-Wilk tests, *p* > 0.05) and were analysed using Welch two-sample *t*-tests.

## Supplementary Information

**Additional file 1: Table S1-S5.** Summary statistics and model output for experiments 1-5; **Table S6.** Cox proportional hazards analysis output for effect of social environment on mating latency; **Table S7.** of social environment on mating duration. **Fig. S1.** Effect of virgin eggs on post-mating fecundity by females held in isolation prior to mating. **Fig. S2.** Effect of social environment on mating latency. **Fig. S3.** Effect of social environment on mating duration.

**Additional file 2.** Raw data.

## Data Availability

All data generated or analysed during this study are included in Additional file 2.

## Abbreviations

ANOVA: Analysis of variance
GLM: Generalised linear model
IQR: Interquartile range
SY: Sugar yeast medium

## References

[1] West-Eberhard MJ (2003). Developmental plasticity and evolution.

[2] Kasumovic MM, Brooks RC (2011). It’s all who you know: the evolution of socially cued anticipatory plasticity as a mating strategy. Q Rev Biol.

[3] Davis JM, Nufio CR, Papaj DR (2011). Resource quality or competition: why increase resource acceptance in the presence of conspecifics?. Behav Ecol.

[4] Huang P, Sieving KE, Mary CMS (2011). Heterospecific information about predation risk influences exploratory behavior. Behav Ecol.

[5] Bretman A, Fricke C, Chapman T (2009). Plastic responses of male *Drosophila melanogaster* to the level of sperm competition increase male reproductive fitness. Proc Biol Sci.

[6] Dore AA, McDowall L, Rouse J, Bretman A, Gage MJG, Chapman T (2018). The role of complex cues in social and reproductive plasticity. Behav Ecol Sociobiol.

[7] Parker GA, Pizzari T (2010). Sperm competition and ejaculate economics. Biol Rev Camb Philos Soc.

[8] Wedell N, Gage MJ, Parker GA (2002). Sperm competition, male prudence and sperm-limited females. Trends Ecol Evol.

[9] Bretman A, Gage MJ, Chapman T (2011). Quick-change artists: male plastic behavioural responses to rivals. Trends Ecol Evol.

[10] Garbaczewska M, Billeter JC, Levine JD (2013). *Drosophila melanogaster* males increase the number of sperm in their ejaculate when perceiving rival males. J Insect Physiol.

[11] Wigby S, Sirot LK, Linklater JR, Buehner N, Calboli FC, Bretman A (2009). Seminal fluid protein allocation and male reproductive success. Curr Biol.

[12] Kawecki TJ (1995). Adaptive plasticity of egg size in response to competition in the cowpea weevil, *Callosobruchus maculatus* (Coleoptera: Bruchidae). Oecologia..

[13] Burns CW (1995). Effects of crowding and different food levels on growth and reproductive investment of Daphnia. Oecologia..

[14] Sarin S, Dukas R (2009). Social learning about egg-laying substrates in fruitflies. Proc Biol Sci.

[15] Duménil C, Woud D, Pinto F, Alkema JT, Jansen I, Van Der Geest AM (2016). Pheromonal cues deposited by mated females convey social information about egg-laying sites in *Drosophila melanogaster*. J Chem Ecol.

[16] Malek HL, Long TAF (2020). On the use of private versus social information in oviposition site choice decisions by *Drosophila melanogaster* females. Behav Ecol.

[17] Wertheim B, Dicke M, Vet LE (2002). Behavioural plasticity in support of a benefit for aggregation pheromone use in *Drosophila melanogaster*. Entomol Exp Appl.

[18] Kacsoh BZ, Bozler J, Ramaswami M, Bosco G. Social communication of predator-induced changes in *Drosophila* behavior and germ line physiology. eLife. 2015;4:e07423. 10.7554/eLife.07423 PMC4456452 25970035

[19] Bailey NW, Zuk M (2008). Acoustic experience shapes female mate choice in field crickets. Proc Biol Sci.

[20] Billeter JC, Jagadeesh S, Stepek N, Azanchi R, Levine JD (2012). *Drosophila melanogaster* females change mating behaviour and offspring production based on social context. Proc Biol Sci.

[21] Filice DCS, Long TAF (2017). Phenotypic plasticity in female mate choice behavior is mediated by an interaction of direct and indirect genetic effects in *Drosophila melanogaster*. Ecol Evol.

[22] Fox RJ, Fromhage L, Jennions MD (2019). Sexual selection, phenotypic plasticity and female reproductive output. Philos Trans R Soc Lond B Biol Sci.

[23] Churchill ER, Dytham C, Bridle JR, Thom MDF (2021). Social and physical environment independently affect oviposition decisions in *Drosophila*. Behav Ecol.

[24] Bretman A, Westmancoat JD, Gage MJ, Chapman T (2011). Males use multiple, redundant cues to detect mating rivals. Curr Biol.

[25] Bretman A, Rouse J, Westmancoat JD, Chapman T (2017). The role of species-specific sensory cues in male responses to mating rivals in *Drosophila melanogaster* fruitflies. Ecol Evol.

[26] Laissue PP, Vosshall LB (2008). The olfactory sensory map in Drosophila. Ad Exp Med Biol.

[27] Larsson MC, Domingos AI, Jones WD, Chiappe ME, Amrein H, Vosshall LB (2004). Or83b encodes a broadly expressed odorant receptor essential for *Drosophila* olfaction. Neuron..

[28] Ferreiro MJ, Pérez C, Marchesano M, Ruiz S, Caputi A, Aguilera P (2017). *Drosophila melanogaster* white mutant w(1118) undergo retinal degeneration. Front Neurosci.

[29] Ashburner M (1989). Drosophila. A laboratory handbook.

[30] Wertheim B, Marchais J, Vet LEM, Dicke M (2002). Allee effect in larval resource exploitation in *Drosophila*: an interaction among density of adults, larvae, and micro-organisms. Ecol Entomol.

[31] Marchini D, Marri L, Rosetto M, Manetti AG, Dallai R (1997). Presence of antibacterial peptides on the laid egg chorion of the medfly *Ceratitis capitata*. Biochem Biophys Res Commun.

[32] Narasimha S, Nagornov KO, Menin L, Mucciolo A, Rohwedder A, Humbel BM (2019). *Drosophila melanogaster* cloak their eggs with pheromones, which prevents cannibalism. PLoS Biol.

[33] Rockwell R, Grossfield J (1978). *Drosophila*: behavioral cues for oviposition. Am Midl Nat.

[34] Markow TA, O’Grady P (2005). *Drosophila*: a guide to species identification and use.

[35] Symonds M, Wertheim B (2005). The mode of evolution of aggregation pheromones in *Drosophila* species. J Evol Biol.

[36] Jaenike J, Bartelt RJ, Huberty AF, Thibault S, Libler JS (1992). Aggregations in mycophagous *Drosophila* (Diptera: Drosophilidae): candidate pheromones and field responses. Ann Entomol Soc Am.

[37] Wertheim B. Ecology of Drosophila aggregation pheromone: a multitrophic approach: PhD Thesis. The Netherlands: Wageningen University; 2001.

[38] Wertheim B (2005). Evolutionary ecology of communication signals that induce aggregative behaviour. Oikos..

[39] Elsensohn JE, Aly MFK, Schal C, Burrack HJ (2021). Social signals mediate oviposition site selection in *Drosophila suzukii*. Sci Rep.

[40] Rouse J, McDowall L, Mitchell Z, Duncan EJ, Bretman A (1935). Social competition stimulates cognitive performance in a sex-specific manner. Proc Biol Sci.

[41] Bretman A, Fricke C, Hetherington P, Stone R, Chapman T (2010). Exposure to rivals and plastic responses to sperm competition in *Drosophila melanogaster*. Behav Ecol.

[42] Rouse J, Bretman A (2016). Exposure time to rivals and sensory cues affect how quickly males respond to changes in sperm competition threat. Anim Behav.

[43] Billeter JC, Wolfner MF (2018). Chemical cues that guide female reproduction in *Drosophila melanogaster*. J Chem Ecol.

[44] Keesey IW, Koerte S, Retzke T, Haverkamp A, Hansson BS, Knaden M (2016). Adult frass provides a pheromone signature for *Drosophila* feeding and aggregation. J Chem Ecol.

[45] Yang C-h, Belawat P, Hafen E, Jan LY, Jan Y-N (2008). *Drosophila* egg-laying site selection as a system to study simple decision-making processes. Science..

[46] Sitaraman D, Zars M, LaFerriere H, Chen Y-C, Sable-Smith A, Kitamoto T (2008). Serotonin is necessary for place memory in *Drosophila*. Proc Natl Acad Sci U S A.

[47] Armstrong JD, Texada MJ, Munjaal R, Baker DA, Beckingham KM (2006). Gravitaxis in *Drosophila melanogaster*: a forward genetic screen. Genes Brain Behav.

[48] Boulétreau-merle J, Fouillet P (2002). How to overwinter and be a founder: egg-retention phenotypes and mating status in *Drosophila melanogaster*. Evol Ecol.

[49] Prokopy RJ, Bush GL (1973). Oviposition by grouped and isolated apple maggot flies. Ann Entomol Soc Am.

[50] Edward DA, Poissant J, Wilson AJ, Chapman T (2014). Sexual conflict and interacting phenotypes: a quantitative genetic analysis of fecundity and copula duration in *Drosophila melanogaster*. Evolution..

[51] Bretman A, Westmancoat JD, Chapman T (2013). Male control of mating duration following exposure to rivals in fruitflies. J Insect Physiol.

[52] Bretman A, Westmancoat JD, Gage MJ, Chapman T (2013). Costs and benefits of lifetime exposure to mating rivals in male *Drosophila melanogaster*. Evolution..

[53] Dore AA, Bretman A, Chapman T (2020). Fitness consequences of redundant cues of competition in male *Drosophila melanogaster*. Ecol Evol.

[54] Bath E, Bowden S, Peters C, Reddy A, Tobias JA, Easton-Calabria E (2017). Sperm and sex peptide stimulate aggression in female *Drosophila*. Nat Ecol Evol.

[55] Tamura K, Subramanian S, Kumar S (2004). Temporal patterns of fruit fly (*Drosophila*) evolution revealed by mutation clocks. Mol Biol Evol.

[56] Broughton SJ, Piper MD, Ikeya T, Bass TM, Jacobson J, Driege Y (2005). Longer lifespan, altered metabolism, and stress resistance in *Drosophila* from ablation of cells making insulin-like ligands. Proc Natl Acad Sci U S A.

[57] Göpfert MC, Robert D (2001). Biomechanics. Turning the key on *Drosophila* audition. Nature..

[58] van Naters WVDG, Carlson JR (2007). Receptors and neurons for fly odors in *Drosophila*. Curr Biol.

[59] Team RC (2013). R: a language and environment for statistical computing.

[60] Gilchrist AS, Partridge L (2000). Why it is difficult to model sperm displacement in *Drosophila melanogaster*: the relation between sperm transfer and copulation duration. Evolution..

