# Supplementary material for: Memory of social experience affects female fecundity via perception of fly deposits
Source: BMC Biol. 2022 Oct 31;20:244. doi: 10.1186/s12915-022-01438-5 (PMC9620669; doi:10.1186/s12915-022-01438-5)
Supplement: Supplementary file 1 — Additional file 1: Table S1-S5. Summary statistics and model output for experiments 1-5; Table S6. Cox proportional hazards analysis output for effect of social environment on mating latency; Table S7. of social environment on mating duration. Fig. S1. Effect of virgin eggs on post-mating fecundity by females held in isolation prior to mating. Fig. S2. Effect of social environment on mating latency. Fig. S3. Effect of social environment on mating duration. [file 12915_2022_1438_MOESM1_ESM.pdf]

Memory of social experience affects female fecundity via perception of fly deposits

Fowler, E.K.[1], Leigh, S.[1], Rostant, W.G.[1], Thomas, A.[1], Bretman, A.[2], Chapman, T.[1,#]

[1]School of Biological Sciences, University of East Anglia, Norwich Research Park, Norwich, NR4 7TJ, UK.

[2]School of Biology, Faculty of Biological Sciences, University of Leeds, Leeds, LS2 9JT, UK.

Phone: + 44 (0)1603 593210

[#]Author for correspondence: Tracey Chapman

e-mail: tracey.chapman@uea.ac.uk

Additional File 1

Table S1. Summary statistics and model output for experiment 1 (Female fecundity responses to variation in the social environment and effect of exposure to con- vs hetero-specific females).

**Experiment 1**						
Summary statistics						
**treatment**	**n**	**mean eggs**	**sd**	**se**	**95% ci**	
isolated	46	36.04	15.54	2.29	4.62	
melanogaster	40	29.43	13.31	2.10	4.26	
simulans	32	28.28	15.43	2.73	5.56	
yakuba	46	23.20	13.32	1.96	3.96	
Model summary: summary(glm(eggs ~ treatment, data, quasipoisson))						
	**Estimate**	**std. error**	**t value**	**p value**		
(Intercept)	3.58	0.07	54.66	<2e-16		
melanogaster	-0.20	0.10	-1.99	0.048 *		
simulans	-0.24	0.11	-2.17	0.031 *		
yakuba	-0.44	0.10	-4.21	4.33e-5 ***		
Analysis of deviance: anova(model,test = "F")						
	**Df**	**Deviance**	**Resid. Df**	**Resid. Dev**	**F**	**p**
NULL			163	1398.7		
treatment	3	130.48	160	1268.2	6.10	0.00059***

Table S2 Summary statistics and model outputs for experiment 2 (Effect of length of social exposure period on post-mating fecundity).

**Experiment 2**						
Summary statistics						
**timepoint**	**social treatment**	**n**	**mean eggs**	**sd**	**se**	**95% ci**
2h	isolated	43	28.8	15.4	2.4	4.8
2h	group	46	31.8	16.1	2.4	4.8
4h	isolated	44	29.6	14.1	2.1	4.3
4h	group	44	30.1	15.4	2.3	4.7
8h	isolated	35	34.1	17.3	2.9	5.9
8h	group	42	30.7	9.5	1.5	3.0
24h	isolated	58	39.4	20.9	2.7	5.5
24h	group	59	37.4	19.0	2.5	4.9
48h	isolated	57	38.5	19.3	2.6	5.1
48h	group	60	32.8	12.4	1.6	3.2
72h	isolated	60	41.8	20.5	2.6	5.3
72h	group	62	28.3	12.3	1.6	3.1
Full model summary – timepoints 2h, 4h and 8h						
	**Estimate**	**std. error**	**t value**	**p value**		
(Intercept)	3.36	0.08	44.39	<2e-16		
treatmentgroup	0.10	0.10	0.96	0.337		
timepoint4	0.03	0.11	0.27	0.790		
timepoint8	0.17	0.10	1.58	0.116		
group:timepoint4	-0.08	0.15	-0.56	0.575		
group:timepoint8	-0.21	0.15	-1.39	0.166		
Analysis of deviance – full model vs model without interaction						
	**Df**	**Deviance**	**Resid. Df**	**Resid. Dev**	**F**	**p**
-interaction			250	2010.5		
+interaction	2	13.82	248	1996.7	0.97	0.379
Posthoc test: emmeans(short_model, ~ pairwise ~ treatment | timepoint)						
	**estimate**	**SE**	**z-ratio**	**p value**		
2h	-0.10	0.10	-0.96	0.34		
4h	-0.02	0.10	-0.16	0.87		
8h	0.11	0.11	1.00	0.32		
Full model summary – timepoints 24h, 48h and 72h						
	**Estimate**	**std. error**	**t value**	**p value**		
(Intercept)	3.67	0.06	60.69	<2e-16		
treatmentgroup	-0.05	0.09	-0.61	0.545		
timepoint48	-0.02	0.09	-0.27	0.784		
timepoint72	0.06	0.08	0.70	0.486		
group:timepoint48	-0.11	0.12	-0.86	0.392		
group:timepoint72	-0.34	0.12	-2.71	0.007 **		
Analysis of deviance – full model vs model without interaction						
	**Df**	**Deviance**	**Resid. Df**	**Resid. Dev**	**F**	**p**
-interaction			352	3602.2		
+interaction	2	63.82	350	3538.4	3.81	0.023*
Posthoc test: emmeans(long_model, ~ pairwise ~ treatment | timepoint)						
	**estimate**	**SE**	**z-ratio**	**p value**		
24h	0.05	0.09	0.61	0.55		

48h	0.16	0.09	1.77	0.08 .		
72h	0.39	0.10	4.33	<0.0001 ***		

Table S3 Summary statistics and model outputs for experiment 3 (Investigation of whether exposure to eggs or to female deposits in the absence of eggs are required for social exposure effects on post-mating fecundity).

**Experiment 3**						
**Part 1: eggless and conditioned vials**						
Summary statistics						
**treatment**	**n**	**mean eggs**	**sd**	**se**	**95% ci**	
isolated	44	36.59	15.69	2.37	4.77	
conditioned	46	27.32	14.82	2.19	4.40	
eggless	35	29.31	15.29	2.58	5.25	
group	39	21.85	10.32	1.65	3.35	
Model summary						
	**Estimate**	**std. error**	**t value**	**p value**		
(Intercept)	3.60	0.07	54.89	<2e-16		
conditioned	-0.29	0.10	-2.95	0.004 **		
eggless	-0.22	0.11	-2.11	0.036 *		
group	-0.52	0.11	-4.63	7.63e-6 ***		
Analysis of deviance – model vs null						
	**Df**	**Deviance**	**Resid. Df**	**Resid. Dev**	**F**	**p**
NULL			163	1355.2		
treatment	3	160.59	160	1194.6	7.73	7.44e-5***
**Part 2: spiked eggs**						
Summary statistics						
**treatment**	**n**	**mean eggs**	**sd**	**se**	**95% ci**	
isolated	39	51.15	17.94	2.87	5.82	
group	39	34.49	14.58	2.33	4.73	
spiked	32	37.41	24.28	4.29	8.75	
Model summary						
	**Estimate**	**std. error**	**t value**	**p value**		
(Intercept)	3.93	0.07	58.62	<2e-16		
group	-0.39	0.11	-3.73	0.0003 ***		
spiked	-0.31	0.11	-2.86	0.0052 **		
Analysis of deviance – model vs null						
	**Df**	**Deviance**	**Resid. Df**	**Resid. Dev**	**F**	**p**
NULL			109	1112.98		
treatment	2	143.75	107	969.23	8.00	0.0006***

Table S4: Summary statistics and model outputs for experiment 4 (Investigation of the sensory pathways required to detect cues of social exposure effects on post-mating fecundity)

**Experiment 4**						
**Part 1: antennaless**						
Summary statistics						
**social treatment**	**line**	**N**	**mean eggs**	**sd**	**se**	**95% ci**
isolated	control	43	34.70	17.53	2.67	5.40
group	control	42	27.50	17.38	2.68	5.42
isolated	antennaless	35	33.23	23.47	3.97	8.06
group	antennaless	30	23.23	17.82	3.25	6.65
Model summary						
	**Estimate**	**std. error**	**t value**	**p value**		
(Intercept)	3.55	0.09	39.20	<2e-16		
treatmentgroup	-0.23	0.14	-1.70	0.092 .		
lineantennaless	-0.04	0.14	-0.32	0.752		
group:antennaless	-0.13	0.22	-0.58	0.563		
Analysis of deviance – full model vs model without interaction						
	**Df**	**Deviance**	**Resid. Df**	**Resid. Dev**	**F**	**p**
-interaction			147	2288.2		
+interaction	1	4.11	146	2284.1	0.34	0.562
Posthoc test: emmeans(ant_mod1, ~ pairwise ~ treatment | line)						
	**estimate**	**SE**	**z-ratio**	**p value**		
control	0.232	0.137	1.70	0.089 .		
antennaless	0.358	0.167	2.14	0.033 *		
**Part 2: Orco1**						
Summary statistics						
**social treatment**	**line**	**N**	**mean eggs**	**sd**	**se**	**95% ci**
isolated	control	48	47.92	18.38	2.65	5.34
group	control	43	40.16	16.05	2.45	4.94
isolated	orco1	32	36.19	14.44	2.55	5.20
group	orco1	38	28.05	13.49	2.19	4.43
Model summary						
	**Estimate**	**std. error**	**t value**	**p value**		
(Intercept)	3.87	0.05	72.83	<2e-16		
treatmentgroup	-0.18	0.08	-2.18	0.031 *		
lineorco1	-0.28	0.09	-3.06	0.003 **		
group:orco1	-0.08	0.14	-0.58	0.564		
Analysis of deviance – full model vs model without interaction						
	**Df**	**Deviance**	**Resid. Df**	**Resid. Dev**	**F**	**p**
-interaction			158	1161.6		
+interaction	1	2.17	157	1159.4	0.33	0.564
Posthoc test: emmeans(orco_mod1, ~ pairwise ~ treatment | line)						
	**estimate**	**SE**	**z-ratio**	**p value**		
control	0.177	0.08	2.18	0.030 *		
orco1	0.255	0.11	2.35	0.019 *		
**Part 3: Tactile**						
Summary statistics						
**social treatment**	**vial setup**	**n**	**mean eggs**	**sd**	**se**	**95% ci**
isolated	control	41	50.34	20.99	3.28	6.62
group	control	42	38.30	15.02	2.32	4.68
isolated	divided	48	39.52	15.20	2.19	4.42

group	divided	38	38.76	15.35	2.49	5.04
Model summary						
	**Estimate**	**std. error**	**t value**	**p value**		
(Intercept)	3.92	0.06	69.23	<2e-16		
socialgroup	-0.27	0.09	-3.19	0.002 **		
vial.setupdivided	-0.24	0.08	-2.96	0.004 **		
group:divided	0.25	0.12	2.05	0.042 *		
Analysis of deviance – full model vs model without interaction						
	**Df**	**Deviance**	**Resid. Df**	**Resid. Dev**	**F**	**p**
-interaction			166	1438.8		
+interaction	1	27.82	165	1411.0	4.21	0.042*
Posthoc tests: emmeans(touch_mod1, ~ pairwise ~ social | vial.setup)						
	**estimate**	**SE**	**z-ratio**	**p value**		
control	0.27	0.086	3.19	0.0014 **		
divided	0.02	0.089	0.22	0.8284		
**Part 4: vision**						
summary statistics						
**social treatment**	**vision treatment**	**N**	**mean eggs**	**sd**	**se**	**95% ci**
isolated	control	43	43.40	14.82	2.26	4.56
group	control	41	29.85	15.68	2.45	4.95
isolated	dark	43	50.91	13.82	2.11	4.25
group	dark	45	38.76	18.39	2.74	5.52
isolated	white	48	56.31	21.10	3.05	6.13
group	white	41	54.34	19.40	3.03	6.12
model summary						
	**Estimate**	**std. error**	**t value**	**p value**		
(Intercept)	3.77	0.06	62.50	<2e-16		
socialgroup	-0.37	0.10	-3.90	0.0001 ***		
socialdark	0.16	0.08	1.94	0.0530 .		
socialwhite	0.26	0.08	3.32	0.0010 **		
socialgroup:visiondark	0.10	0.13	0.80	0.4266		
socialgroup:vision white	0.34	0.12	2.79	0.0057 **		
Analysis of deviance – full model vs model without interaction						
	**Df**	**Deviance**	**Resid. Df**	**Resid. Dev**	**F**	**p**
-interaction			257	1880.5		
+interaction	2	60.57	255	1819.9	4.46	0.012 *
posthoc test: emmeans(vision_mod1, ~ pairwise ~ social_treatment | vision_treatment)						
	**estimate**	**SE**	**z-ratio**	**p value**		
control	0.374	0.096	3.90	0.0001 ***		
dark	0.273	0.084	3.26	0.0011 **		
white	0.036	0.075	0.48	0.6328		

Table S5 Summary statistics and model outputs for experiment 5 (Effect of social environment on virgin egg retention)

**Experiment 5**						
Summary statistics for virgin egg data						
**treatment**	**day**	**n**	**mean virgin eggs**	**sd**	**se**	**95% ci**
alone	1	50	7.52	12.83	1.81	3.65
group	1	50	10.94	10.12	1.43	2.88
alone	2	49	3.94	7.28	1.04	2.09
group	2	48	3.98	5.59	0.81	1.62
alone	3	49	2.53	5.15	0.74	1.48
group	3	44	6.86	7.81	1.18	2.37
**Effect of social treatment and day of social exposure on virgin eggs**						
Model summary: hurdle(virgin_eggs ~ treatment*day, data = long_virgin, dist = "negbin")						
Count model coefficients (truncated negbin with log link)						
	**Estimate**	**std. error**	**z value**	**p value**		
(Intercept)	3.09	0.16	19.49	<2e-16 ***		
treatmentgroup	-0.43	0.19	-2.25	0.024 *		
day2	-0.61	0.23	-2.63	0.009 **		
day3	-0.93	0.25	-3.77	0.0002 ***		
group:2	0.14	0.30	0.47	0.636		
group:3	0.64	0.30	2.12	0.034 *		
Log(theta)	0.96	0.17	5.69	1.25e-08 ***		
Zero hurdle model coefficients (binomial with logit link)						
	**Estimate**	**std. error**	**z value**	**p value**		
(Intercept)	-0.66	0.30	-2.22	0.026		
treatmentgroup	1.82	0.45	4.07	4.6e-05		
day2	-0.06	0.43	-0.14	0.887		
day3	-0.25	0.43	-0.58	0.561		
group:2	-1.34	0.61	-2.19	0.029 *		
group:3	-0.34	0.63	-0.54	0.589		
Analysis of deviance – full model vs model without interaction (wald test)						
	**res. Df**	**Df**	**χ^2**	**p value**		
-interaction	281					
+interaction	277	4	9.95	0.041 *		
posthoc test: emmeans(mod.hurdle.nb, ~ pairwise ~ treatment | day, mode = "zero")						
	**estimate**	**SE**	**t-ratio**	**p value**		
day 1	-0.425	0.091	-4.69	<0.0001 ***		
day 2	-0.116	0.100	-1.17	0.24		
day 3	-0.354	0.100	-3.58	0.0004 ***		
posthoc test: emmeans(mod.hurdle.nb, ~ pairwise ~ treatment | day, mode = "count")						
	**estimate**	**SE**	**t-ratio**	**p value**		
day 1	7.77	3.84	2.03	0.04 *		
day 2	3.02	2.47	1.22	0.22		

day 3	-1.97	2.16	-0.90	0.36		
**Effect of virgin eggs on post-mating eggs**						
Model summary: glm(pmeggs ~ virgin_eggs + social_treatment, data, family = "quasipoisson")						
	**Estimate**	**std. error**	**t value**	**p value**		
(Intercept)	3.77	0.060	62.40	<2e-16		
virgin eggs	-0.01	0.003	-4.48	2.35e-05 ***		
socialtreatmentgroup	-0.23	0.090	-2.64	0.010 *		
Analysis of deviance						
	**Df**	**Deviance**	**Resid. Df**	**Resid. Dev**	**F**	**p**
NULL			87	652.6		
virgin eggs	1	148.2	86	504.5	30.9	<0.0001***
socialtreatment	1	33.8	85	470.7	7.1	0.009**

Table S6. Cox proportional hazards analysis output for effect of social environment on mating latency for the control group across eight experiments in this study. Mating latency was analysed using a cox proportional hazards test with censorship and the number of censored points included in the overall sample size are indicated in brackets with an asterisk.

**Experiment**	**n**	**z**	**p-value**	**lower 95% CI**	**upper 95% CI**
A (baseline)	I = 47 (1*)<br>G = 40 (4*)	-2.03	0.04 *	0.42	0.99
B (exposure length)	I = 61<br>G = 62 (1*)	-4.36	1.32e-05 ***	0.29	0.63
C (Sudan red)	I = 46 (2*)<br>G = 41 (1*)	-2.96	0.0031 **	0.34	0.80
D (egg spiked)	I = 39<br>G = 39	-3.20	0.0014 **	0.27	0.73
E (eggless)	I = 44 (6*)<br>G = 40 (7*)	-1.64	0.10	0.45	1.07
F (antennaless)	I = 44 (2*)<br>G = 42 (4*)	-0.89	0.38	0.54	1.26
G (visual)	I = 43 (2*)<br>G = 42 (2*)	1.05	0.29	0.83	1.93
H (tactile/gustatory)	I = 42 (2*)<br>G = 42 (2*)	-0.60	0.55	0.57	1.35
I (*orco1*)	I = 48 (2*)<br>G = 42 (5*)	-3.38	0.00072 ***	0.31	0.73

Table S7. Effect of social environment on mating duration for the control group across eight experiments in this study. Output from Welch's two-sample t-test. The final sample size is given, following the removal of outlying points if applicable (durations of < 6 minutes and > 30 minutes). The numbers of outlying points removed, if any, are indicated in brackets.

**Experiment**	**n**	**mean duration (minutes)**	**t**	**df**	**p-value**	**lower 95% CI**	**upper 95% CI**
A (baseline)	I = 46 (1)<br>G = 37 (3)	I = 17.9<br>G = 16.8	1.62	78.9	0.11	-0.25	2.44
B (exposure length)	I = 61<br>G = 62	I = 18.6<br>G = 17.3	2.08	120.5	0.04 *	0.06	2.48
C (Sudan red)	I = 46<br>G = 39 (2)	I = 16.5<br>G = 17.1	-1.03	82.9	0.31	-1.84	0.58
D (egg spiked)	I = 39<br>G = 39	I = 18.5<br>G = 18.6	-0.22	74.8	0.83	-1.81	1.45
E (eggless)	I = 44<br>G = 40	I = 17.7<br>G = 17.8	-0.08	80.2	0.94	-1.77	1.63
F (antennaless)	I = 44<br>G = 41 (1)	I = 19.5<br>G = 18.7	0.91	81.7	0.36	-0.91	2.45
G (visual)	I = 43<br>G = 42	I = 20.4<br>G = 19.1	1.78	81.8	0.08	-0.15	2.75
H (tactile/gustatory)	I = 39 (3)<br>G = 42	I = 16.4<br>G = 17.4	-1.40	79.0	0.16	-2.59	0.45
I (*orco1*)	I = 48<br>G = 41 (1)	I = 17.7<br>G = 18.6	-1.26	86.5	0.21	-2.37	0.53

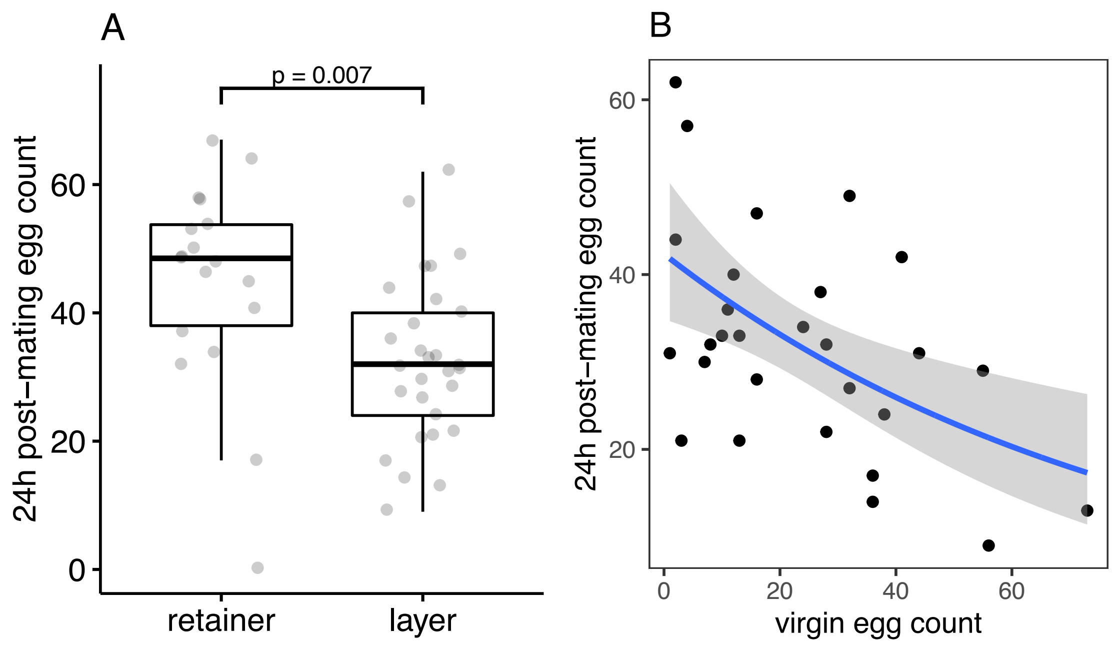

Figure S1. Effect of number of virgin eggs laid over three days on post-mating fecundity by females held in isolation prior to mating. (A) Post-mating egg count was compared for females defined as layers (≥ 1 virgin egg laid) and retainers (zero virgin eggs laid) ($F_{(1,45)} = 7.91$, $p = 0.007$). (B) Relationship between virgin and post-mating eggs for layers only ($F_{(1,27)} = 10.94$, $p = 0.003$).

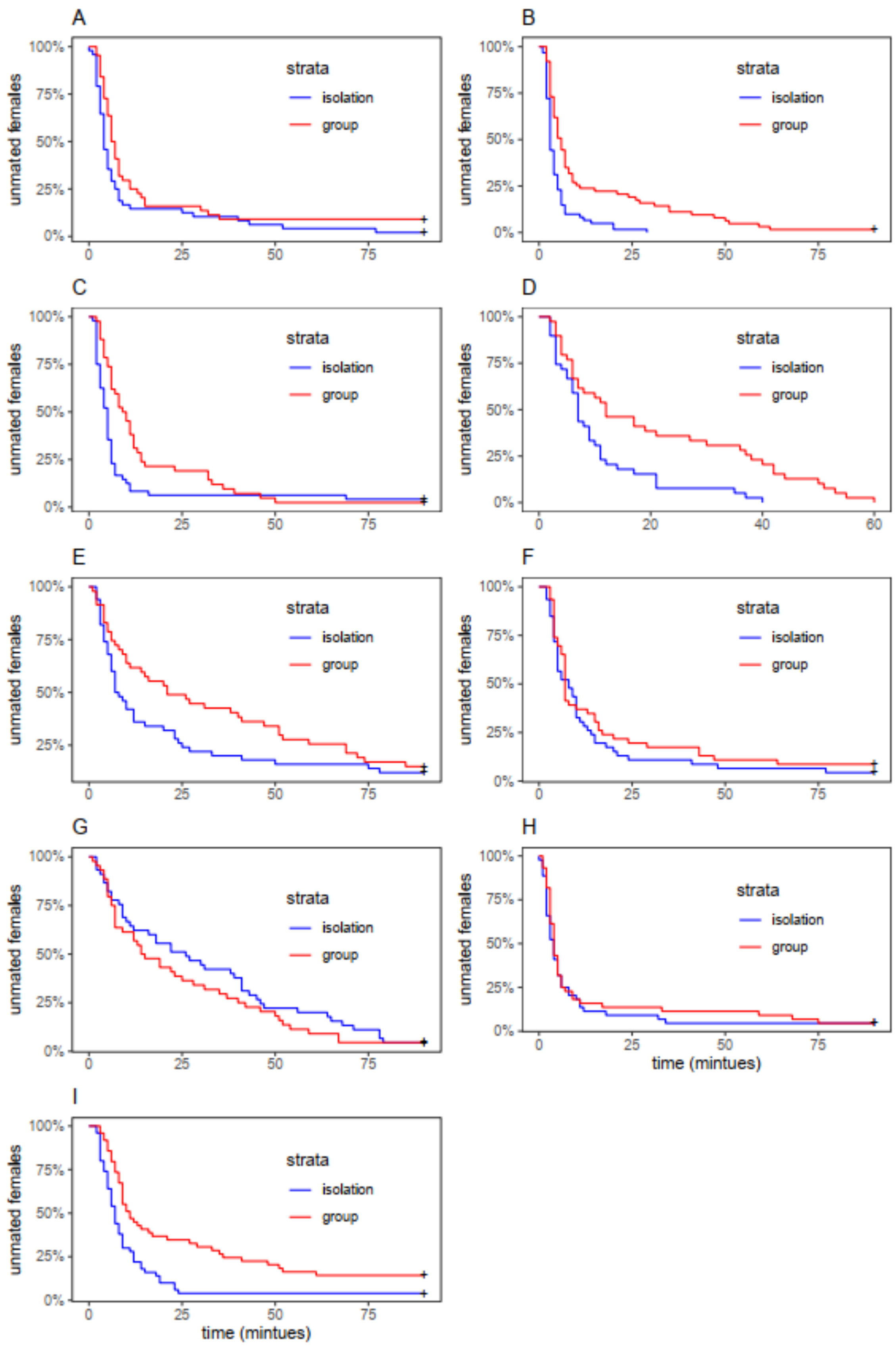

Figure S2. Effect of social environment on mating latency across eight separate experiments in this study. Females were either kept in isolation (blue) or housed in groups of four (red) for 72 hours prior

to mating. The experiments were: (A) baseline responses (Experiment 1 in the main text); (B) Effect of length of exposure to pre-mating social environment, 72 hr timepoint only (Experiment 2 in main text); (C) Effect of social environment on virgin eggs (Experiment 6 in main text); (D) control from the “egg-spiked” block (Experiment 4 in main text); (E) control from the “OvoD1” block (Experiment 4 in main text); (F) control from the antennaless experiment; (G) control from the visual cues experiment; (H) control from the tactile cues experiment; (I) control from the *Orco1* experiment (all Experiment 5 in main text).

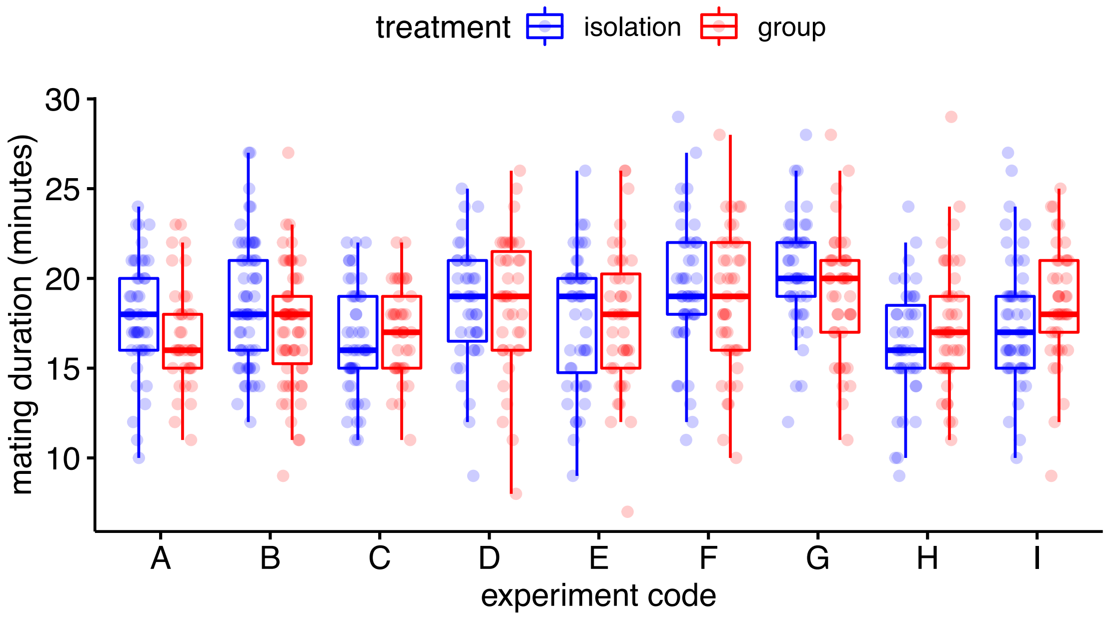

Figure S3. Mating duration of females kept in social-isolation or in a group prior to mating across eight separate experiments in this study. For each experiment, only the data for control flies was analysed to enable direct comparison between experiments (i.e. all data are for intact, wildtype *D. melanogaster* females set up according to the standard experiment protocol). The experiments were: (A) baseline responses (Experiment 1 in the main text); (B) Effect of length of exposure to pre-mating social environment, 72 hr timepoint only (Experiment 2 in main text); (C) Effect of social environment on virgin eggs (Experiment 6 in main text); (D) control from the “egg-spiked” block (Experiment 4 in main text); (E) control from the “OvoD1” block (Experiment 4 in main text); (F) control from the antennaless experiment; (G) control from the visual cues experiment; (H) control from the tactile cues experiment; (I) control from the *Orco1* experiment (all Experiment 5 in main text).
